# Supplementary material for: Single-Cell Virology: On-Chip, Quantitative Characterization of the Dynamics of Virus Spread from One Single Cell to Another
Source: Viruses. 2024 Oct 24;16(11):1659. doi: 10.3390/v16111659 (PMC11598947; doi:10.3390/v16111659)
Supplement: Supplementary file 1 [file viruses-16-01659-s001.zip › Liu et al VIRUSES 2024 SI.pdf]

Supplemental Information

Single-cell virology: On-chip, quantitative characterization of the dynamics of virus spread from one single cell to another

Table of content:

Figure S1 Design of the microfluidic device. (S2)

Figure S2 siRNA knockdown of LC3. (S3)

Table S1 P-values between groups based on t-test for the donor cells. (S4)

Table S2 P-values between groups based on t-test for the donor cells upon treatment of 1 μM pocapavir. (S5)

Movie S1 No spread observed from infected cell to recipient cell

Movie S2 Lytic spread observed from infected cell to recipient cell

Movie S3 Non-lytic spread observed from infected cell to recipient cell

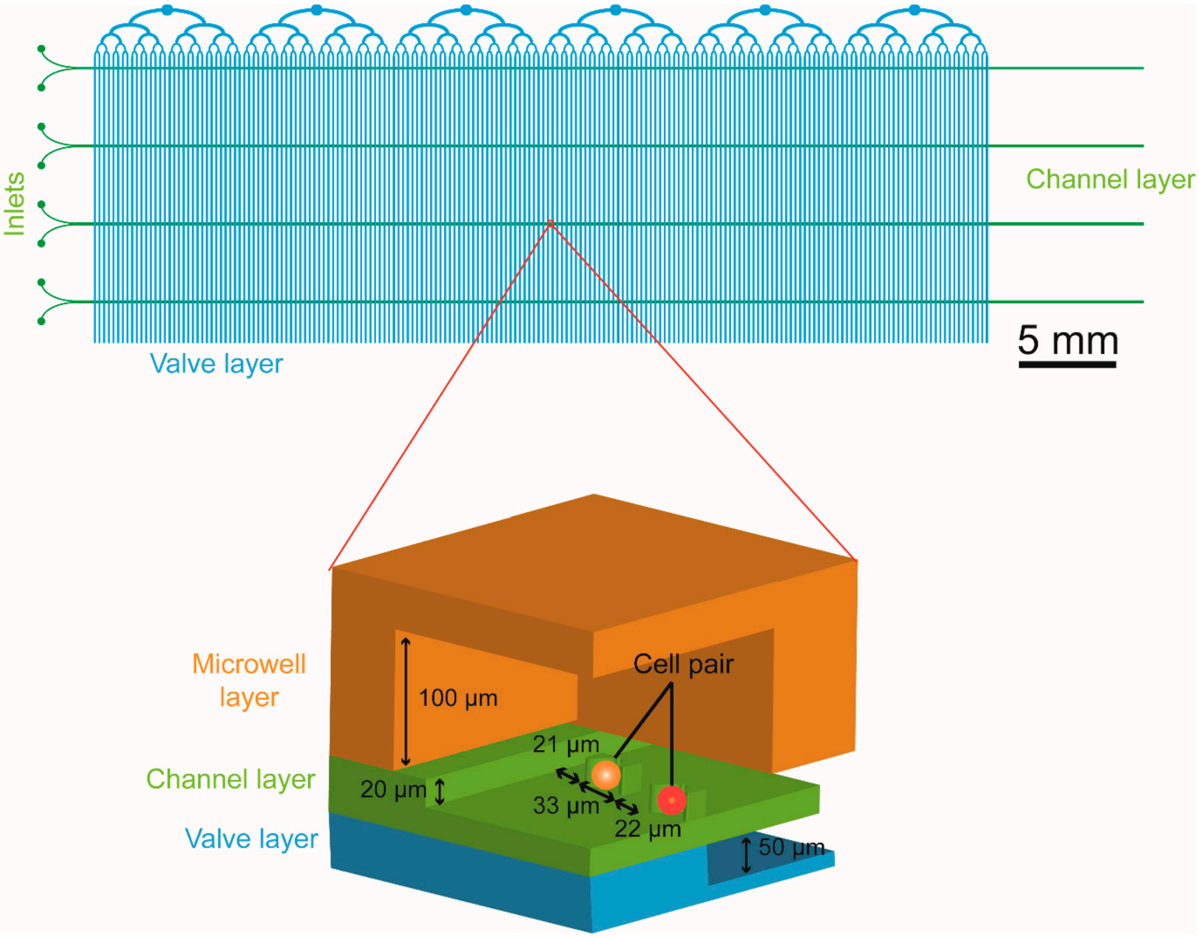

Figure S1. Design of the microfluidic device.

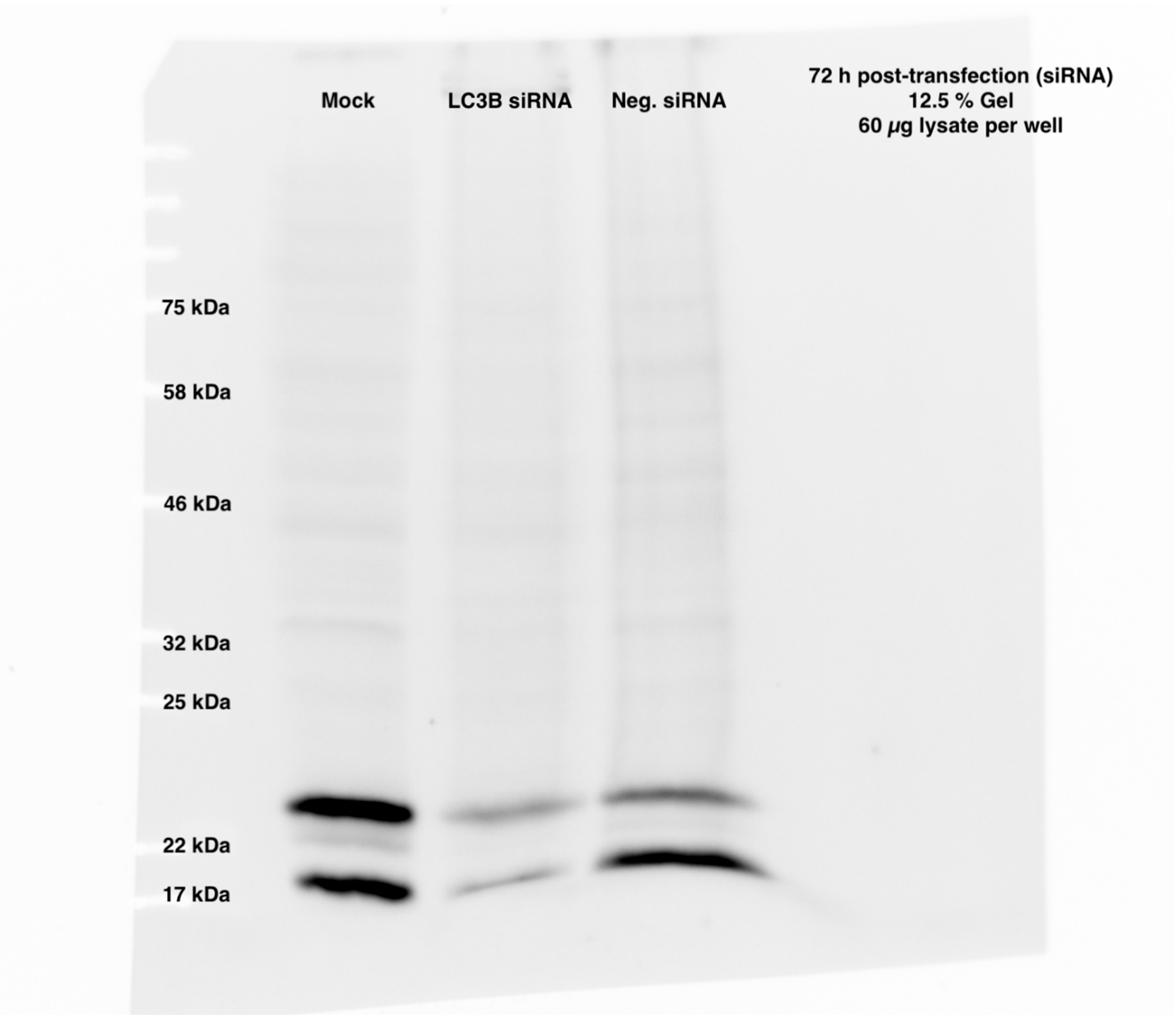

Figure S2. siRNA knockdown of cellular LC3. Protein lysates of HeLa S3 cells were prepared 72 h following treatment with mock, non-targeting or LC3 siRNA.

Table S1 P-values between groups based on t-test for the donor cells.

	Group	No spread	Lytic spread	Nonlytic spread
**Maximum**	No spread	1	0.139548	0.968321
	Lytic spread	0.139548	1	0.372561
	Nonlytic spread	0.968321	0.371561	1
**Slope**	Group	No spread	Lytic spread	Nonlytic spread
	No spread	1	**0.031805**	**0.025294**
	Lytic spread	**0.031805**	1	0.947469
	Nonlytic spread	**0.025294**	0.947469	1
**Infection time**	Group	No spread	Lytic spread	Nonlytic spread
	No spread	1	**0.029273**	**0.013869**
	Lytic spread	**0.029273**	1	0.651774
	Nonlytic spread	**0.013869**	0.651774	1
**Start point**	Group	No spread	Lytic spread	Nonlytic spread
	No spread	1	**0.000016**	0.658761
	Lytic spread	**0.000016**	1	**0.000285**
	Nonlytic spread	0.658761	**0.000285**	1

Table S2 P-values between groups based on t-test for the donor cells upon treatment of 1 μM pocapavir.

	Group	No spread	Lytic spread	Nonlytic spread
**Maximum**	No spread	1	**0.001286**	0.911859
	Lytic spread	**0.001286**	1	**0.006431**
	Nonlytic spread	0.911859	**0.006431**	1
**Slope**	Group	No spread	Lytic spread	Nonlytic spread
	No spread	1	**< 0.00001**	**0.035631**
	Lytic spread	**< 0.00001**	1	**0.006827**
	Nonlytic spread	**0.035631**	**0.006827**	1
**Infection time**	Group	No spread	Lytic spread	Nonlytic spread
	No spread	1	**0.006166**	**0.031876**
	Lytic spread	**0.006166**	1	0.222806
	Nonlytic spread	**0.031875**	0.222806	1
**Start point**	Group	No spread	Lytic spread	Nonlytic spread
	No spread	1	**0.018326**	0.504608
	Lytic spread	**0.018326**	1	0.054253
	Nonlytic spread	0.504608	0.054253	1
